# Spatial relationship between *Taenia solium* tapeworm carriers and necropsy cyst burden in pigs

**DOI:** 10.1371/journal.pntd.0005536

**Published:** 2017-04-13

**Authors:** Ian W. Pray, Viterbo Ayvar, Ricardo Gamboa, Claudio Muro, Luz M. Moyano, Victor Benavides, Robert H. Flecker, Hector H. Garcia, Seth E. O’Neal

**Affiliations:** 1 School of Public Health, Oregon Health & Science University and Portland State University, Portland, Oregon, United States of America; 2 Center for Global Health Tumbes, Universidad Peruana Cayetano Heredia, Tumbes, Peru; 3 School of Sciences, Department of Microbiology, Universidad Peruana Cayetano Heredia, Lima, Peru; George Washington University School of Medicine and Health Sciences, UNITED STATES

## Abstract

**Background:**

*Taenia solium*, a parasite that affects humans and pigs, is the leading cause of preventable epilepsy in the developing world. Geographic hotspots of pigs testing positive for serologic markers of *T*. *solium* exposure have been observed surrounding the locations of human tapeworm carriers. This clustered pattern of seropositivity in endemic areas formed the basis for geographically targeted control interventions, which have been effective at reducing transmission. In this study, we further explore the spatial relationship between human tapeworm carriers and infected pigs using necroscopic examination as a quantitative gold-standard diagnostic to detect viable *T*. *solium* cyst infection in pigs.

**Methodology/Principal findings:**

We performed necroscopic examinations on pigs from 7 villages in northern Peru to determine the number of viable *T*. *solium* cysts in each pig. Participating humans in the study villages were tested for *T*. *solium* tapeworm infection (i.e., taeniasis) with an ELISA coproantigen assay, and the distances from each pig to its nearest human tapeworm carrier were calculated. We assessed the relationship between proximity to a tapeworm carrier and the prevalence of light, moderate, and heavy cyst burden in pigs. The prevalence of pig infection was greatest within 50 meters of a tapeworm carrier and decreased monotonically as distance increased. Pigs living less than 50 meters from a human tapeworm carrier were 4.6 times more likely to be infected with at least one cyst than more distant pigs. Heavier cyst burdens, however, were not more strongly associated with proximity to tapeworm carriers than light cyst burdens.

**Conclusion/Significance:**

Our study shows that human tapeworm carriers and pigs with viable *T*. *solium* cyst infection are geographically correlated in endemic areas. This finding supports control strategies that treat humans and pigs based on their proximity to other infected individuals. We did not, however, find sufficient evidence that heavier cyst burdens in pigs would serve as improved targets for geographically focused control interventions.

## Author summary

*Taenia solium*, the pork tapeworm, is a parasite transmitted between humans and pigs. The disease is most common in developing countries where access to sanitation is limited and domestic pigs are allowed to roam freely. Humans infected with the intestinal tapeworm release *T*. *solium* eggs into the environment when they defecate outside, and pigs become infected with the larval stage of the disease, cysticercosis, upon consuming these eggs in human feces. Prior work using serologic testing of pigs has shown that proximity to human tapeworm carriers is a possible risk factor for porcine cysticercosis. Our study investigated if proximity to human tapeworm carriers was associated with established cyst infection in pigs, and if proximity to tapeworm carriers also increased the degree of cyst infection in pigs (i.e., the number of *T*. *solium* cysts a pig was infected with). The results confirmed that human tapeworm carriers and infected pigs are geographically correlated, but did not uncover a stronger relationship for heavily infected pigs. It is important to continue to investigate this spatial relationship as some control strategies rely on the assumption that human tapeworm carriers will be found in close proximity to heavily infected pigs. Future control strategies would benefit from a precise knowledge of the degree of pig infection that best predicts for nearby tapeworm carriers.

## Introduction

*Taenia solium*, the pork tapeworm, is a parasite that affects 50 million people worldwide [1]. When the parasite infects the human central nervous system, the result is a severe neurological condition called neurocysticercosis (NCC), which may lead to seizures, headaches, and stroke. In Latin America alone, 1.3 million people have epilepsy from NCC [2], and, in rural Peru, 1 in 200 people suffer from epilepsy caused by NCC [3].

*T*. *solium* is transmitted between humans and pigs, and is commonly found in rural areas of low income countries where access to sanitation is limited and free-roaming pigs have access to human feces. An adult tapeworm residing in the human gut produces millions of infectious eggs over its lifespan that are expelled through the feces of the infected human host (a condition called taeniasis). When infected humans defecate outside, *T*. *solium* eggs may be consumed by free-ranging pigs and develop into larval cysts that lodge in the soft tissue of the pigs (a condition called porcine cysticercosis). Humans may, in turn, be infected with the intestinal tapeworm by consuming these cysts in undercooked pork.

Transmission of the *T*. *solium* parasite varies considerably by location, as significant variations in prevalence have been observed both at a regional scale [3–6], and among households within a community [7,8]. Detecting these spatial patterns of *T*. *solium* infection, whether on a regional scale or at the household level, is an important step in the development of effective control strategies. The most common spatial pattern analysis that has been used to study *T*. *solium* has been the detection of clusters of a single type of infection (e.g., porcine cysticercosis). To this end, studies in both Latin America [4,9] and Africa [7,10] have found that cases of porcine cystiercosis tend to be clustered within the same households and among neighboring households within the same communities. Such studies that identify univariate clusters of infection are important first steps in understanding disease distribution, and may be used to prioritize the allocation of scarce resources for prevention.

Other studies have sought to examine these clusters of cysticercosis in relation to the locations of human tapeworm carriers as potential sources of infection. Examining the precise spatial relationship between human and porcine hosts allows for the investigation of physical and biologic mechanisms dictating *T*. *solium* transmission, which can then be used to design spatially explicit control strategies. In separate studies conducted in endemic regions of Peru, Garcia et al. [4] and Lescano et al. [11] found that living in the same household as a tapeworm carrier was an important risk factor for cysticercosis seropositivity among humans. Similarly, clusters of porcine cysticercosis have been found to occur in hotspots surrounding human tapeworm carriers. Lescano et al. found that pigs living less than 50 meters from a tapeworm carrier were much more likely to be seropositive than more distant pigs [12], and O’Neal et al. found that the prevalence of human taeniasis was significantly increased among individuals residing within 100 meters of an infected pig [13].

The results of these latest distance analyses directly led to the development of a control methodology known as “ring strategy”. Ring strategy targets anti-helminthic treatment to only those humans and pigs that reside within 100 meters of a positively identified pig. This targeted approach to treatment was developed as an alternative to mass anti-helminthic treatment in Peru, and was based on the assumption that pig and human disease are spatially dependent and likely be found in close proximity to each other. Ring interventions have now been trialed in endemic communities of Peru, and have shown early success, with significant reductions in pig seroincidence observed in intervention communities [14].

The strong associations observed between cysticercosis infection and tapeworm carriers in previous spatial analyses, together with the early success of ring strategies, suggests that location and proximity are important determinants of *T*. *solium* transmission. Despite this knowledge, important gaps remain in our understanding of the spatial dynamics of *T*. *solium* transmission that impede our ability to understand transmission mechanisms, and improve upon existing control strategies. First, most studies that have investigated the spatial association between human tapeworm carriers and infected pigs have relied on testing pig sera for the presence of antibodies against *T*. *solium*, which does not distinguish active cyst infection from cleared infection or exposure to *T*. *solium* eggs without infection [15]. Necroscopic examination of pigs, which provides a count of the total number of viable *T*. *solium* cysts in pigs, is the most sensitive and specific diagnostic currently available for *T*. *solium* cyst infection in pigs, and would allow us to draw more confident conclusions about the spatial relationships that have been observed. In addition, previous studies have been limited to only assessing the presence or absence of pig infection based on serologic markers. Counting the total number of *T*. *solium* cysts in necropsied pigs provides a quantitative measure of the degree of infection. A spatial analysis of cyst burden would allow us to detect a biologic gradient between the degree of infection (i.e., number of cysts counted on pig necropsy) and their proximity to a tapeworm carrier. This association could provide important insight into the environmental and biologic mechanisms driving *T*. *solium* egg dispersion and cyst infection, and may lead to the identification of more specific diagnostic targets (e.g., pigs of a specific cyst burden) for ring strategies.

In order fill these knowledge gaps we performed a distance analysis examining the relationship between *T*. *solium* cyst infection in pigs and their distance to infected human tapeworm carriers in an endemic region of northern Peru. Specifically, we assessed this spatial relationship at different burdens of cyst infection and at different distances. Our objectives were to determine if pigs with heavier cyst burdens were more likely to be found in close proximity to tapeworm carriers, and to determine if a critical distance threshold could be identified at which the relationship between human tapeworm carriers and infected pigs could no longer be observed. Based on the positive findings of previous studies, we hypothesized that a strong association between infection in pigs and their proximity to human tapeworm carriers would exist, and would become stronger at heavier burdens of infection.

## Methods

Data for this study were collected in 2015 as part of a trial testing a community-driven ring treatment strategy for the control of *T*. *solium* in 7 villages of northern Peru. By the time data for this study were collected, pigs in these communities had not received antiparasitic treatment for at least 9 months, and the human population had not yet been intervened upon. At the conclusion of the trial, all humans were offered testing and treatment for taeniasis, and seropositive pigs were purchased and euthanized for necroscropic examination of cyst burden.

### Human participants

We performed a door-to-door survey of all households in the 7 villages and attempted to recruit all human residents older than 2 years of age for participation. Consenting participants were interviewed for household and demographic characteristics. Survey questions included the age and sex of each pig, the presence and condition of a pig corral on the property, the source of household drinking water, and human waste disposal (open field defecation or latrine). We used handheld GPS receivers (GeoExplorer II; Trimble, Sunnyvale, CA) with post-processed differential correction for sub-meter accuracy to record a single set of coordinates in front of each household. These coordinates were used to represent the locations of both human and pig participants in each household. At the conclusion of the trial period, all participants were presumptively treated for taeniasis with a single oral dose of niclosamide according to their weight (11–34 kg received 1 g; 35–50 kg received 1.5 g; > 50 kg received 2 g), and were instructed to collect their next stool. Niclosamide was chosen for mass treatment because it is highly effective against taeniasis [16,17], and does not affect the cystic stage of *T*. *solium* like other available chemotherapies, which could cause neurological symptoms in undiagnosed cases of NCC [18]. Post-treatment stool samples were first tested with enzyme-linked immunosorbent assays for *T*. *solium* coproantigens (CoAg-ELISA) as previously described [19]. Reactive samples (optical density ratio (ODR) > 7.5%) were examined microscopically for the presence of *Taenia* spp. eggs in stool using the test tube spontaneous sedimentation technique [20], and humans with reactive samples were followed up after two weeks with further testing and treatment to confirm clearance. Other intestinal parasites that were detected during stool screening were provided appropriate treatment through the local health center. For this analysis, we considered humans to be positive for *T*. *solium* taeniasis if *Taenia* spp. eggs were visualized in stool or the CoAg-ELISA test produced an ODR greater than 20%. We choose to use ODR > 20% as a case definition in this analysis to reduce the rate of false positives due to non-specific binding and cross-reaction with other *Taenia spp*., which may occur at low ODR values [21,22].

### Swine participants and necropsy

All pigs older than four weeks of age were eligible for participation in this study. Serum samples were collected from all eligible pigs in the study villages at the conclusion of the year-long trial, and were analyzed by enzyme-linked immunoelectrotransfer blot (EITB) to detect the presence serum antibodies that indicate exposure to *T*. *solium* eggs. Briefly, the EITB assay measures reactivity of pig serum to seven lentil-lectin purified glycoprotein antigens isolated from native cysts [23]. Reaction to 1 or more of these glycoprotein antigens bands is highly sensitive for detecting active cyst infection (89%), however lacks specificity (48%); while reaction to 4 or more bands is less sensitive (61%), but has improved specificity (92%) [15]. Given that the expected prevalence of active cyst infection among pigs in this region of Peru is around 5–10%, the predictive value of a negative EITB assay is high (Garcia et al. found that 99% (144 out of 146) of seronegative pigs in this region were necropsy-negative [17]).

Results from the EITB assay were used to select pigs for necroscopic examination. In order to prevent the unnecessary sacrifice of uninfected pigs, we attempted to purchase only pigs with one or more positive EITB bands for necropsy. Pigs with negative serologic results were assumed to contain zero cysts, as negative EITB results are highly predictive of negative necropsy results [17]. Of the 791 pigs tested from the seven study villages, 419 (53%) seropositive pigs were identified. Study staff attempted to purchase all seropositive pigs for necroscopic examination, however, due to reluctance of villagers to sell their animals, only 146 (35%) of these seropositive pigs were able to be purchased. Purchased pigs were anesthetized and humanely euthanized. To determine the number of viable *T*. *solium* cysts in each necropsied pig, the entire carcass was dissected and systematically inspected using fine tissue slices of less than 0.5 cm. Viable cysts were those with well-delineated thin-walled cystic structures containing clear vesicular fluid and a visible white protoscolex, however a formal bile test was not conducted to confirm viability. Degenerated and calcified cysts, while enumerated by examiners, were not included in this analysis. For pigs with particularly dense cyst burdens, a weighed sample of forelimb muscle was counted for cysts and extrapolated to estimate the total body burden.

Our final analysis was carried out on a sample of 515 pigs (65% of 791 total pigs). This sample was composed of the 146 (28%) seropositive pigs that study staff purchased from pig-owners for necroscopic examination and 369 (72%) seronegative pigs for which a cyst count of zero was imputed. The remaining 272 serepositive pigs in our sample were excluded because necroscopic examination was not performed and cyst burden could not be estimated.

### Statistical analysis

In ArcMap 10.3 (ESRI; Redland, CA), we plotted the household locations of study participants (humans and pigs) using a transverse Mercator projection (UTM Peru 17S, 1996). We then calculated the Euclidean distance in meters from each pig’s household to the nearest human tapeworm carrier household. Pigs living in the same household as a human tapeworm carrier were given a distance value of zero. Distances were categorized into bins of < 50 meters, 50–500 meters and > 500 meters. These groupings were chosen because they produced a well-delineated gradient of infection prevalence at increasing distances. The 100 meter distance threshold was not included in our results because no effect was observed among pigs 50–100 meters from a tapeworm carrier.

Due to the lack of normality in the dependent variable (cyst burden), we elected to categorize this variable into bins based on the following schema: heavy infection (≥ 100 viable cysts), moderate infection (10–99 viable cysts), light infection (1–9 viable cysts) and no infection (zero viable cysts or negative EITB serology). We used logistic regression models with binary outcomes to examine predictors for three different cyst burden thresholds (≥ 1 cyst, ≥ 10 cysts, and ≥ 100 cysts), using pigs with no infection (zero viable cysts or negative EITB serology) as a reference group in all models. Logistic regression models with robust sandwich estimators from the generalized estimating equations (GEE) family were used to account for household clustering (i.e., dependence between pigs from the same household). We first created bivariate models for pig- and household-level predictors and selected covariates to include in our final multivariable models if they were significant (α = 0.05) in any of the three cyst burden models.

### Ethics

This study was reviewed and approved at Oregon Health and Science University, Portland, Oregon, USA, by the Institutional Review Board (protocol #10116) and the Institutional Animal Care and Use Committee (protocol #2843). It was also reviewed and approved at Universidad Peruana Cayetano Heredia, Lima, Peru, by the Institutional Ethics Committee (protocol #61326), and the Institutional Committee for the Ethical Use of Animals (protocol #61326). Written informed consent was obtained from all human participants. The consent of an adult or guardian was required for the participation of children <18 years old. Treatment of animals adhered to the Council for International Organizations of Medical Sciences (CIOMS) International Guiding Principles for Biomedical Research Involving Animals. Pigs were humanely euthanized by administering 0.1 mg/kg of xylazine with 5 mg/kg of ketamine intravenously to achieve deep anesthesia, followed by injection of 100 mg/kg of sodium pentobartital.

## Results

### Human population

The 7 participating villages ranged in population from 130 to 596 human inhabitants, for a total population of 1,890 individuals (Table 1). 32% of the population reported practicing open field defecation, and 63% of the population reported raising pigs. In total, 1,420 (75%) participants submitted stool samples for parasite testing. Residents who declined to submit stool samples were more likely to be male, younger, and practice open defecation (S1 Table). A geographic analysis of participating and non-participating households across the 7 study villages revealed no concerning spatial patterns of non-participation (Ripley’s K1-K2 test for random labelling [24,25], S1 Appendix).

**Table 1:** Human and pig characteristics by village.

	Culqui (1)	Cachaco (2)	B. Aires (3)	La Saucha (4)	P. Quiroz (5)	Algodonal (6)	Tom. Cardal (7)	Total
**HUMAN POPULATION**								
Human population size	596	130	160	280	273	211	240	**1890**
Practice of open field defecation	126 (21%)	23 (18%)	5 (3%)	88 (31%)	84 (31%)	102 (48%)	179 (75%)	**607 (32%)**
Submitted stool sample	448 (75%)	92 (71%)	132 (83%)	214 (76%)	218 (80%)	151 (72%)	165 (69%)	**1420 (75%)**
*T*. *solium* taeniasis (prevalence)†	9 (2.0%)	1 (1.1%)	6 (4.6%)	4 (1.9%)	2 (0.9%)	8 (5.3%)	4 (2.4%)	**34 (2.4%)**
ELISA Co-Ag ODR ≥ 20%	7 (78%)	1 (100%)	6 (100%)	4 (100%)	2 (100%)	8 (100%)	4 (100%)	**32 (94%)**
*Taenia spp*. eggs in stool	8 (89%)	1 (100%)	1 (17%)	2 (50%)	2 (100%)	3 (38%)	4 (100%)	**21 (62%)**
**SWINE POPULATION**								
Pig population size	255	101	55	109	100	85	86	**791**
EITB-LLGB serology								
Seronegative (0 bands)	129 (51%)	63 (63%)	20 (36%)	47 (43%)	56 (56%)	30 (35%)	27 (31%)	**372 (47%)**
≥ 1 positive band	126 (49%)	38 (38%)	35 (64%)	62 (57%)	44 (44%)	55 (65%)	59 (69%)	**419 (53%)**
≥ 4 positive bands	24 (9%)	1 (1%)	8 (15%)	12 (11%)	5 (5%)	6 (7%)	17 (20%)	**74 (9%)**
**Swine necropsy and characteristics**								
Total pigs included in distance analysis	174	77	34	76	72	38	44	**515**
Necroscopic examination	45 (26%)	14 (18%)	14 (41%)	30 (39%)	16 (22%)	10 (26%)	17 (39%)	**146 (28%)**
Seronegative*	129 (74%)	63 (82%)	20 (59%)	46 (61%)	56 (78%)	28 (74%)	27 (61%)	**369 (72%)**
Age in months (median, IQR)	8 (5–12)	6 (4–9)	10 (8–12)	8 (6–12)	9 (7–12)	5 (3–8)	9 (6.5–10)	**8 (5–11)**
Sex, female	87 (50%)	46 (60%)	17 (50%)	37 (49%)	39 (54%)	25 (66%)	25 (57%)	**276 (54%)**
Meters to nearest *T*. *solium* tapeworm carrier (median, IQR)	164 (41–232)	407 (345–788)	184 (106–318)	308 (222–399)	1059 (749–1191)	41 (0–94)	462 (383–713)	**271 (113–462)**
*T*. *solium* cysticercosis infection								
≥ 1 cyst (any infection)	13 (7.5%)	2 (2.6%)	3 (8.8%)	7 (9.2%)	4 (5.6%)	5 (13.2%)	10 (22.7%)	**44 (8.5%)**
≥ 10 cysts (moderate-heavy)	5 (2.9%)	0 (0%)	3 (8.8%)	4 (5.3%)	0 (0%)	1 (2.6%)	5 (11.4%)	**18 (3.5%)**
≥ 100 cysts (heavy)	2 (1.2%)	0 (0%)	0 (0.0%)	2 (2.6%)	0 (0.0%)	1 (2.6%)	5 (11.4%)	**10 (1.9%)**

†Positive identification of *Taenia* spp. eggs in stool or optical density ratio (ODR) ≥ 20% in ELISA coproantigen assay

*3 seronegative pigs excluded from distance analysis because they were geographic outliers

Among human participants, 34 (2.4%) showed evidence of a *T*. *solium* taeniasis. The prevalence of taeniasis ranged from 0.9% to 5.3% among the seven study villages. Of the 34 taeniasis cases, 26 (76%) tested positive on CoAg-ELISA with ODR ≥ 40%, while 6 (18%) had CoAg-ELISA ODR between 20% and 40%, and 2 (6%) were diagnosed by microscopy alone (ODR < 20%). A sensitivity analysis showing the possible impact of these different case definitions on the observed associations is presented in S2 Appendix.

### Swine serology

Serum samples were collected from all eligible pigs in the study villages (n = 791 pigs). Overall, 53% tested positive for antibodies against *T*. *solium* cyst (at least one positive EITB band), and seropositivity ranged from 38% to 69% among the seven study villages (Table 1). 9% of the pigs had 4 or more positive bands, and the prevalence of 4 or more bands ranged from 1% to 20% among the study villages.

### Swine characteristics and necropsy

The 515 pigs included in this study consisted of 146 (28%) pigs that were necropsied, and 369 (72%) seronegative pigs for which a cyst count of zero was imputed. Among study pigs, the median age was 8 months and 54% were female. In terms of *T*. *solium* cyst burden, 471 (92%) of the study pigs were uninfected, 26 (5%) pigs had light infection (1–9 cysts), 8 (2%) pigs had moderate infection (10–99 cysts), and 10 (2%) pigs had heavy infection (≥100 cysts).

### Distance and cyst burden

**Fig 1:**
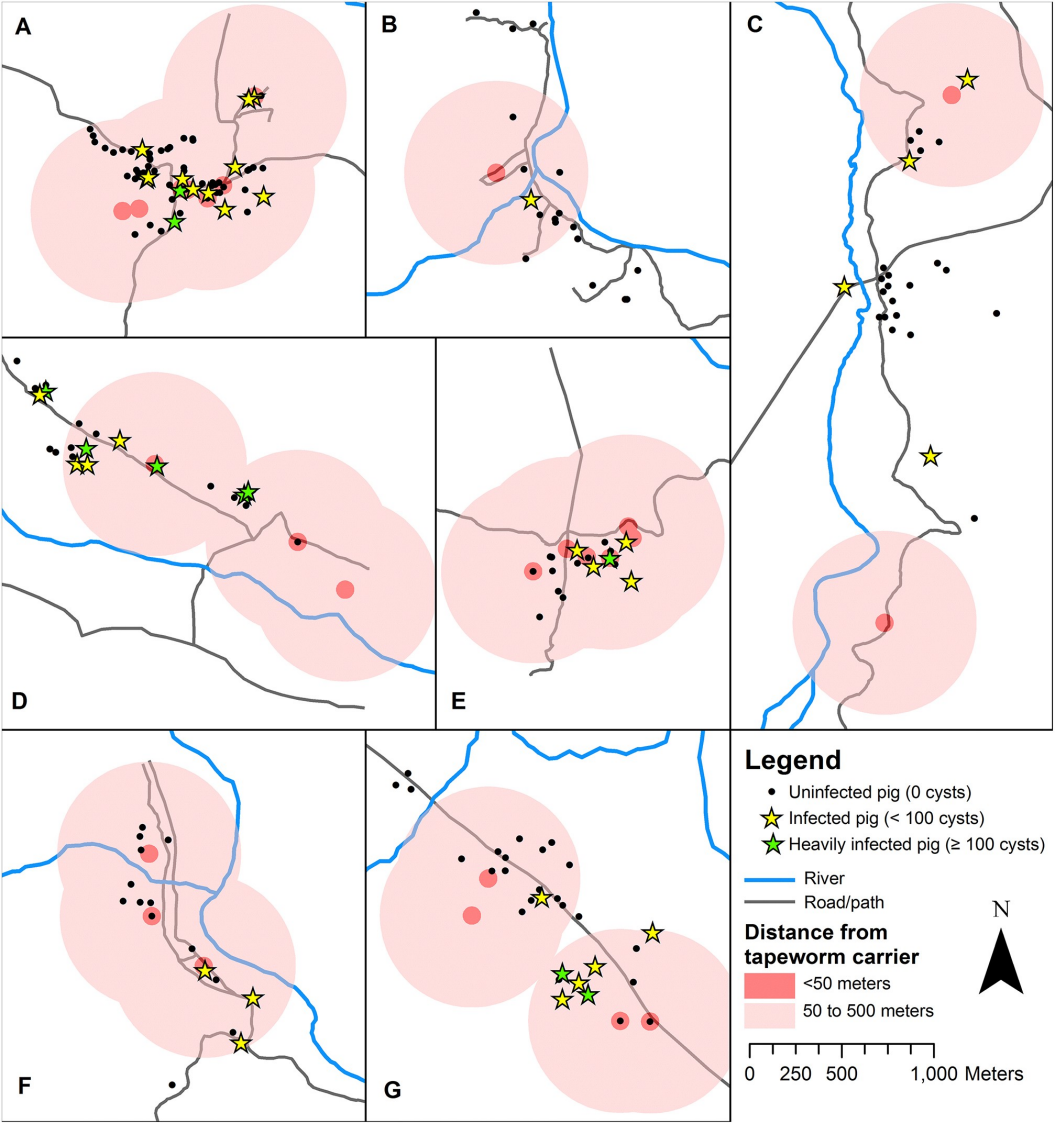
Map of *Taenia solium* tapeworm carriers and infected pigs in study villages. (A) Culqui, (B) Cachaco, (C) Puente Quiroz, (D) Tomopampa de Cardal, (E) Algodonal, (F) Buenos Aires, (G) La Saucha.

**Fig 2:**
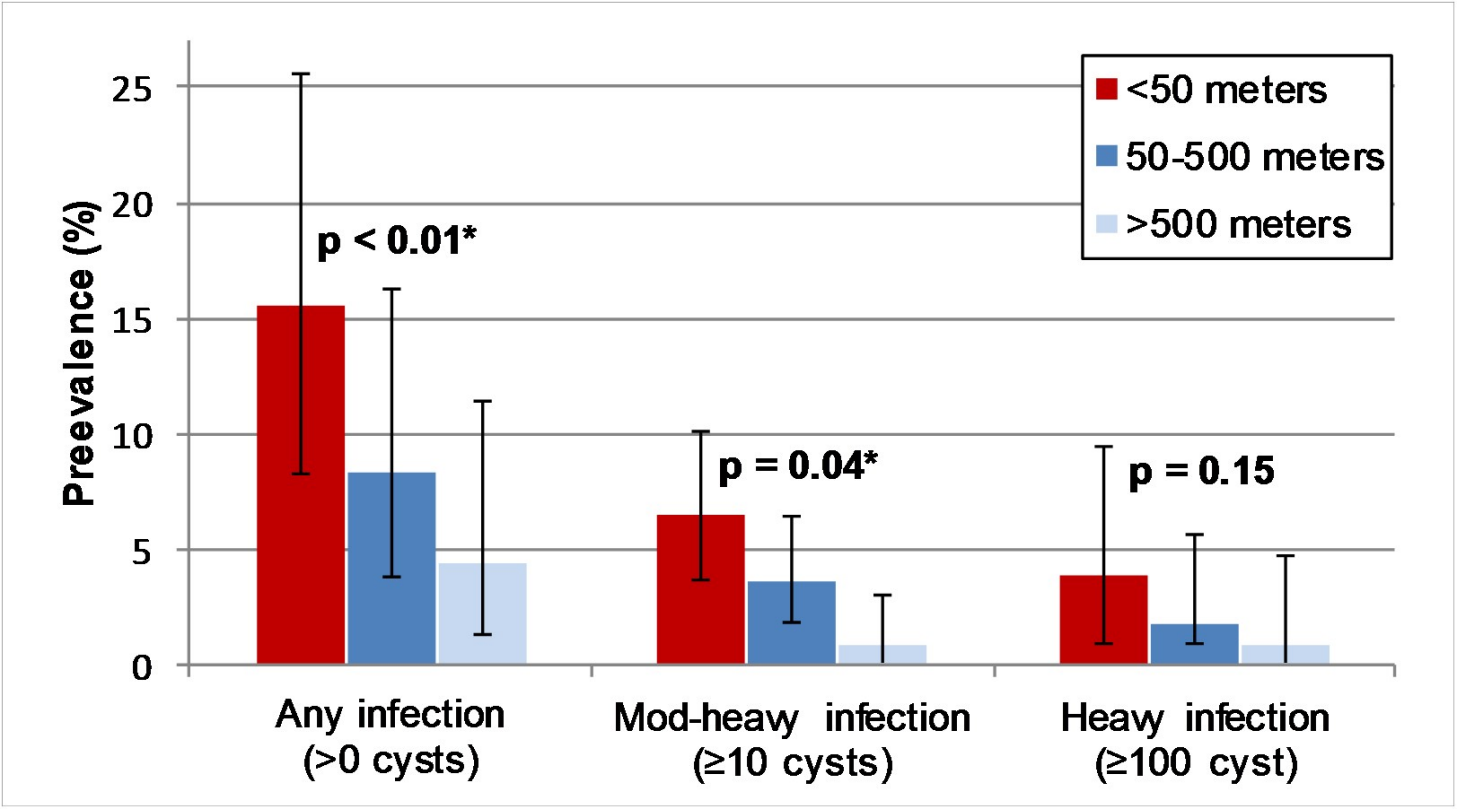
Prevalence of *Taenia solium* infection identified by necropsy (≥ 1, ≥ 10, and ≥ 100 viable cysts) among pigs at increasing distances from human tapeworm carriers. P-value indicates results of chi-square test for distance trend within each cyst burden stratum. 95% confidence bands calculated with exact binomial distribution.

Fig 1 shows the geographical distribution of human tapeworm carriers and infected pigs in the study villages. The prevalence of *T*. *solium* cysticercosis (at least one viable cyst on necropsy) was greatest among pigs living within 50 meters of a tapeworm carrier, and decreased proportionally at greater distances. The prevalence of at least one viable cyst was 15.6% (12 out of 77) at < 50 meters from a tapeworm carrier, 8.3% (27 out of 325) between 50 and 500 meters, and 4.4% (5 out of 113) at > 500 meters (p < 0.01 for trend) (Fig 2). Of the 12 infected pigs living within 50 meters of a tapeworm carrier, 3 (25%) pigs resided in the same household as the tapeworm carrier. Overall, the prevalence of *T*. *solium* infection among pigs owned by tapeworm carriers was 12.5% (3 out of 24). This was not significantly different from the prevalence of pig infection among all pigs living within 50 meters of a tapeworm carrier. The prevalence of moderate-to-heavy cyst infection (≥ 10 viable cysts) and heavy infection cyst infection (≥ 100 viable cysts) showed similar trends of increasing prevalence at closer distances to tapeworm carriers, however the distance trend for heavy infection was non-significant. The prevalence of pigs with ≥ 10 viable cysts was 6.5% (5 out of 77) at < 50 meters, 3.7% (12 out of 325) between 50 and 500 meters, and 0.9% (1 out of 113) at > 500 meters (p = 0.04 for trend), while the prevalence of heavy infection (≥100 viable cysts) was 3.9% (3 out of 77) at < 50 meters, 1.8% (6 out of 325) between 50 and 500 meters, and 0.9% (1 out of 113) at > 500 meters (p = 0.15 for trend).

### Logistic regression

When examined in bivariate logistic regression, only two predictors, distance to the nearest human tapeworm carrier and the age of the pig, were significantly associated with cyst infection. Pigs residing within 50 meters of a tapeworm carrier were significantly more likely to be infected than pigs living more than 500 meters from a tapeworm carrier (Table 2). This association increased in strength between pigs with at least one viable cyst (OR = 4.6; 95% CI: 1.4, 15.4) and 10 or more viable cysts (OR = 8.7, 95% CI: 1.0, 76.1). The 50 meter distance threshold, however, was not significant when tested for heavily infected pigs (100 or more cysts). Similarly, residing in the same households as a tapeworm carrier did not significantly increase the odds of *T*. *solium* cyst infection, regardless of the cyst burden tested. Pigs residing 50 to 500 meters from tapeworm carriers did not have a significantly greater odds of infection (light, moderate, or heavy infection) compared to pigs residing more than 500 meters from a tapeworm carrier (OR = 1.99 for ≥ 1 viable cyst, 95% CI: 0.64, 6.2). Variables that were not significant and thus excluded from the final multivariable model were household defecation practice (latrine versus open field), water source, number of human occupants, the number of pigs owned, the presence of tapeworm carriers in the household, pig sex and the presence of a corral for the pig.

**Table 2:** Crude associations between select pig characteristics and cyst burden (n = 515 pigs).

	Any infection (≥1 cyst)	Moderate-heavy infection (≥ 10 cysts)	Heavy infection (≥ 100 cysts)
**Number of infected pigs**	44 (8.5%)	18 (3.5%)	10 (1.9%)
**Distance to nearest *T*. *solium* tapeworm carrier**			
< 50 meters	4.57 (1.36, 15.4)^	8.72 (1.00, 76.0)^	4.54 (0.47, 43.6)
50–500 meters	1.99 (0.64, 6.2)	3.54 (0.45, 28.2)	2.00 (0.23, 17.3)
> 500 meters	Ref	Ref	Ref
**Pig owner is a *T*. *solium* tapeworm carrier**			
Yes	1.63 (0.37, 7.2)	2.97 (0.69, 12.8)	2.29 (0.31, 16.8)
No	Ref	Ref	Ref
**Treatment of feces by pig owner**			
Open field defecation	1.34 (0.60, 3.03)	0.59 (0.13, 2.74)	1.25 (0.24, 6.57)
Latrine/indoor bathroom	Ref	Ref	Ref
**Corral built for pig**			
Yes	0.84 (0.40, 1.76)	0.93 (0.29, 2.93)	1.31 (0.26, 6.7)
No	Ref	Ref	Ref
**Pig sex**			
Male	0.60 (0.32, 1.12)	0.61 (0.25, 1.51)	0.72 (0.23, 2.25)
Female	Ref	Ref	Ref
**Pig age, per additional month**	1.08 (1.04, 1.12)^	1.08 (1.03, 1.13)^	1.08 (1.01, 1.14)^

Odds ratios from GEE logistic regression model to adjust for household clustering

^p-value < 0.05

Based on our findings from the bivariate analysis, only distance to nearest tapeworm carrier and pig age were included in the final adjusted GEE logistic regression models (Table 3). After adjusting for pig age, pigs living less than 50 meters from a human tapeworm carrier were 4.56 times (95% CI: 1.33, 15.6) more likely to be infected with at least one cyst than pigs living more than 500 meters from a tapeworm carrier. In the two models that assessed the effect of distance at heavier cyst burdens, we found strong but non-significant effects of living less than 50 meters from a tapeworm carrier (OR = 7.27, p = 0.07 for ≥10 cysts; OR = 4.25, p = 0.21 for ≥ 100 cysts). Similar to our findings in the unadjusted analysis, distances greater than 50 meters from a tapeworm carrier, including pigs living 50 to 100 meters from a tapeworm carrier, were not significantly associated with increased pig infection at any cyst burden.

**Table 3:** Multivariable regression of distance to tapeworm carriers and different cyst burdens in pigs.

	Any infection (≥1 cyst)	Moderate-heavy infection (≥ 10 cysts)	Heavy infection (≥ 100 cysts)
**Distance to nearest *T*. *solium* tapeworm carrier**			
<50m	4.56 (1.33, 15.6)^	7.27 (0.87, 61.0)	4.25 (0.44, 41.1)
50-500m	1.95 (0.63, 6.04)	3.01 (0.40, 22.6)	1.89 (0.23, 15.8)
>500m	Ref	Ref	Ref

Odds ratios from GEE logistic regression model adjusted for household clustering and pig age

^p-value < 0.05

The distance bins of <50 meters, 50–500 meters, and >500 meters from a tapeworm carrier were chosen for logistic regression models above because of the strong positive association we found among pigs residing <50 meters from a tapeworm carrier. In order to compare our results with previously trialed ring interventions, which initiated targeted interventions within 100 meters of infected pigs [13,14], we also evaluated the odds of cyst infection among pigs living < 100 meters from a tapeworm carrier. We found that pigs residing <100 meters from a tapeworm carrier had a significantly increased odds of cyst infection compared to pigs living > 500 meters from a tapeworm carrier (OR = 3.54, 95%: 1.09, 11.6); however this association was driven by the strong association among pigs residing < 50 meters from tapeworm carriers, and was not significant for moderate or heavy cyst burdens. Overall, there were few infected pigs residing 50 to 100 meters from tapeworm carriers (8%, 3 out of 36 pigs), and pigs residing only in the distance band of 50 to 100 meters from tapeworm carriers did not have significantly increased odds of infection compared to the reference distance of >500 meters (OR = 2.01, 95% CI: 0.31, 12.9) (S2 Table).

## Discussion

In this analysis, we investigated the association between *T*. *solium* cysts burden and proximity to human tapeworm carriers in villages of northern Peru where *T*. *solium* is endemic. There were a few key findings to highlight in our analysis. First, consistent with our hypothesis, the locations of human tapeworm carriers and pigs infected with viable *T*. *solium* cysts were geographically correlated in the study communities. Prevalence of *T*. *solium* cysticercosis decreased monotonically as distance from a human tapeworm carrier increased (15.6%, 8.3%, and 4.4% for pigs living < 50 meter, 50–500 meters, and > 500 meters from a tapeworm carrier, respectively). Our second hypothesis was that proximity to human tapeworm carriers would show a stronger association with pig infection when examined at heavier cyst burdens, thus representing a gradient effect between distance and cyst burden. However, the only statistically significant association observed in the final adjusted models was the comparison of all infected pigs (at least one cyst) with uninfected pigs. At moderate (≥ 10 cysts) and heavy (≥ 100 cysts) cyst burdens, where we expected to find stronger associations, we found that the associations with proximity to human tapeworm carriers became non-significant. Therefore, we were unable to detect any significant biologic gradient between the burden of infection and proximity to tapeworm carriers. Finally, we found that distances less than 50 meters from human tapeworm carriers were associated with an increased prevalence of viable *T*. *solium* cyst infection in pigs. Pigs living less than 50 meters from a human tapeworm carrier were 4.6 times more likely to be infected with at least one cyst than pigs living more than 500 meters from a tapeworm carrier. Pigs living more than 50 meters from a tapeworm carrier, including pigs living between 50 and 100 meters from a tapeworm carrier, did not have an increased odds of infection at any cyst burden analyzed.

These findings are consistent with a previous study that examined the effect of proximity to human tapeworm carriers on the prevalence of pig seropositivity (as measured by EITB serology) in a similar rural region of Peru [12]. Lescano et al. found that the prevalence of *T*. *solium* seropositivity in pigs decreased as distance from a human tapeworm carrier increased (69%, 36%, and 18% among pigs living < 50 meters, 50–500 meters, and > 500 meters from a tapeworm carrier, respectively). Additionally, they concluded that the 50 meter areas surrounding human tapeworm carriers represented significant foci of transmission, with pigs living in these rings 3.7 times more likely to be seropositive than pigs living more than 500 meters from a tapeworm carrier. Our study, therefore, contributes additional evidence that pigs living within 50 meters from a human tapeworm carrier in this region are at increased risk for *T*. *solium* infection, and uses the gold-standard diagnostic for pig infection to demonstrate the proclivity for tapeworm carriers to shed infectious *T*. *solium* eggs in the areas immediately surrounding their homes.

Neither our study nor previous work provide evidence that distances greater than 50 meters (e.g., 100 meter rings used in ring strategies) are associated with an increased risk of *T*. *solium* infection. While a distance gradient was observed in both our study and the study referenced above, neither found a significant independent effect of distances greater than 50 meters on pig infection. O’Neal et al. found that 100 meter rings represented significant foci of *T*. *solium* transmission in rural Peru; however, this study did not specifically evaluate 50 meter rings to determine which distance was responsible for the increased level of transmission [13]. The idea that 50 meters is a critical distance at which pigs are exposed to increased risk of *T*. *solium* infection in this region is consistent with our understanding of pig range and behavior. A GPS tracking study of pigs in rural Peru found that pigs spent an average of 70% of their time within 50 meters of their residence and interacted with human defecation areas nearly 30 minutes per day inside these 50 meters rings (compared to just 7 minutes per day outside of 50 meters) [26]. Based on these findings, we propose that 50 meter rings accurately represent *T*. *solium* transmission foci in endemic areas of rural Peru.

Our finding that the association between proximity to tapeworm carriers and pig infection did not strengthen at heavier cyst burdens was unexpected. While we observed a strong gradient of infection among heavily infected pigs (prevalence of heavy infection was 3.9%, 1.8%, and 0.9% at < 50 meters, 50–500 meters, and > 500 meters, respectively), we expected to also find an increase in the strength of the proximity effect at higher cyst burdens. The independent effect of proximity to tapeworm carriers on the odds of moderate (≥ 10 cysts) and heavy (≥ 100 cysts) infection, however, were not statistically significant. There are a few possible explanations for this unexpected finding. First, it is possible that pigs living in close proximity to tapeworm carriers are, in fact, exposed to greater concentrations of *T*. *solium* eggs in their residential environments, but that the burden of established cyst infection in pigs is mediated by host factors such as differential immune responses, rather than being driven purely by exposure dose. It is also possible that the lack of an observed dose-response distance effect in this analysis could simply be explained by the small numbers of infected pigs that were represented in our sample. Only 1.9% (10 out of 515) of pigs in our sample were heavily infected, and 3.5% (18 out of 515) had more than 10 cysts. These low cell counts likely made it difficult to observe a significant effect in these groups.

Although distance to human tapeworm carriers was an important predictor for *T*. *solium* infection among pigs, many infected pigs in our study did not reside in close proximity to a tapeworm carrier. In fact, only 27% (12 out of 44) of the infected pigs in this study lived within 50 meters of a tapeworm carrier, and some infected pigs lived more than 1 km from it’s the nearest identified tapeworm carrier. There are a number of possible explanations for this unexpected finding that should be investigated with future studies. First, due to the cross-sectional nature of this study, we were only able to detect prevalent cases of porcine cysticercosis, meaning that cyst infection in older pigs could have been caused by previously treated or recovered tapeworm carriers that were not detected. This could have caused pigs with older infections to appear further from tapeworm carriers than they were at the time of infection. It is also possible that pigs appearing distant from tapeworm carriers were infected through egg dispersion mechanisms, such as dung beetles and flies, which have been identified as possible mechanical vectors capable of dispersing *T*. *solium* eggs over long distances [27–31]. Finally, it is possible that we did not identify all tapeworm carriers in the study area. 25% of human inhabitants did not provide stool specimens for testing, which could represent a significant number of undetected human tapeworm carriers, and could explain the appearance of large distance values for some pigs.

There are a few additional limitations to our study that must be noted. First, while the CoAg-ELISA is the most sensitive and specific diagnostic available to detect human taeniasis [22], cross-reaction with other *Taenia* species and non-specific binding of the CoAg-ELISA assay with host factors are known to occur [21,22]. Therefore, it is possible that our use of the CoAg-ELISA assay for *T*. *solium* tapeworm detection could have allowed for false positive diagnoses, which may have diluted the observed spatial relationship. Our sensitivity analysis, however, found that this scenario is unlikely to have occurred. Additionally, our use of household coordinates to represent human and pig locations could have misrepresented the true location of transmission events. For example, it is possible that some free-roaming pigs in our study were infected by consuming infected human feces distant from their household location. Previous studies of human and pig behavior in this region, however, have shown that pigs tend to roam in close proximity to their owner’s homes, and that open human defecation areas are concentration near household locations [26], suggesting that transmission events are most likely to occur in the immediate proximity of household locations used in this study. Finally, in order to reduce unnecessary animal sacrifice, we imputed cyst burdens of zero for seronegative pigs without performing full necropsies on these animals. Based on our knowledge of the sensitivity of EITB serologic tests [15,17], it is possible that a small proportion of these seronegative pigs were truly infected, likely biasing our estimates towards observing weaker associations.

Cysticercosis has a substantial economic and health burden on populations in endemic rural areas of Peru. The results of this study provide an important first step in understanding the spatial dynamics of cysticercosis infection to support the use of ring strategy in Peru. In order to advance control efforts, however, more research must be done to improve diagnostic tests and improve our understanding of factors affecting the transmission of *T*. *solium* between humans and pigs. Answering these questions and optimizing ring strategy could lead to profound reductions in the burden of cysticercosis and ultimately contribute to elimination in the region.

## Supporting information

S1 Table Demographic comparison of participants and non-participants (human). (DOCX)

S2 Table Crude associations between cyst burden in pigs and alternative distances from tapeworm carriers (n = 515 pigs). (DOCX)

S1 Appendix Spatial analysis of clustering among of participants and non-participants (human). (DOCX)

S2 Appendix Sensitivity analysis comparing distance associations using different case definitions for CoAg-ELISA positivity (human). (DOCX)
