# Supplementary material for: Spatial relationship between Taenia solium tapeworm carriers and necropsy cyst burden in pigs
Source: PLoS Negl Trop Dis. 2017 Apr 13;11(4):e0005536. doi: 10.1371/journal.pntd.0005536 (PMC5404875; doi:10.1371/journal.pntd.0005536)
Supplement: S1 Table — (DOCX) [file pntd.0005536.s001.docx]

**S1 Table. Demographic comparison of participants and non-participants (human).** Participants submitted a stool sample for detection of taeniasis with ELISA coproantigen assay, while non-participants either refused or were unavailable for testing after multiple visits by field staff.

|  | **Participants**  **(n=1420, 75%)** | **Non-participants (n=470, 25%)** | **p-value*** |
| --- | --- | --- | --- |
| **Sex** |  |  | <0.001 |
| Male | 680 (48%) | 316 (67%) |  |
| Female | 740 (52%) | 154 (33%) |  |
|  |  |  |  |
| **Feces** |  |  |  |
| Open defecation | 440 (31%) | 167 (39%) | 0.001 |
| Latrine | 979 (69%) | 257 (61%) |  |
|  |  |  |  |
| **Pig owners** | 924 (65%) | 273 (58%) | 0.006 |
|  |  |  |  |
| **Age (mean [95% CI])** | 32.5 (31.3, 33.7) | 28.7 (26.7, 30.7) | 0.001 |
|  |  |  |  |
| **Number of rooms per household** | 3.74 (3.67, 3.81) | 3.46 (3.29, 3.62) | 0.002 |

*Chi-square test of proportions used for categorical variables, t-test used to compare means of continuous variables.

.
