# Supplementary material for: Spatial relationship between Taenia solium tapeworm carriers and necropsy cyst burden in pigs
Source: PLoS Negl Trop Dis. 2017 Apr 13;11(4):e0005536. doi: 10.1371/journal.pntd.0005536 (PMC5404875; doi:10.1371/journal.pntd.0005536)
Supplement: S2 Table — (DOCX) [file pntd.0005536.s002.docx]

**S2 Table. Crude associations between cyst burden in pigs and alternative distances from tapeworm carriers (n=515 pigs).**

|  | **Any infection**  **(≥1 cyst)** | **Moderate-heavy infection (≥ 10 cysts)** | **Heavy infection**  **(≥ 100 cysts)** |
| --- | --- | --- | --- |
| Number of infected pigs | 44 (8.5%) | 18 (3.5%) | 10 (1.9%) |
| Distance to nearest *T. solium* tapeworm carrier |  |  |  |
| < 50 meters | **4.42 (1.32, 14.8)^** | **8.72 (1.00, 76.0)^** | 4.54 (0.47, 43.6) |
| 50-100 meters | 2.01 (0.31, 12.9) | Null^†^ | Null^†^ |
| *<100 meters* | **3.54 (1.09, 11.6)^** | 5.41 (0.63, 46.9) | 3.02 (0.31, 29.1) |
| 100-200 meters | 1.09 (0.29, 4.11) | 2.08 (0.19, 22.9) | 1.06 (0.06, 17.5) |
| 200-500 meters | 2.52 (0.78, 8.17) | 5.45 (0.66, 45.2) | 3.06 (0.34, 27.6) |
| >500 meters | Ref | Ref | Ref |

Odds ratios from GEE logistic regression model to adjust for household clustering

^p-value < 0.05

^†^No infected pigs in the indicated distance stratum
