# Supplementary material for: Spatial relationship between Taenia solium tapeworm carriers and necropsy cyst burden in pigs
Source: PLoS Negl Trop Dis. 2017 Apr 13;11(4):e0005536. doi: 10.1371/journal.pntd.0005536 (PMC5404875; doi:10.1371/journal.pntd.0005536)

**S1 Appendix. Spatial analysis of clustering among of participants and non-participants (human).**

Objective: To detect differences in the geographic distribution of participating humans (those who submitted stool samples for CoAg-ELISA detection of taeniasis) and non-participants. Non-random spatial patterns in participation could bias our estimates if non-participants were at lesser or greater risk of transmission because of their location within the village.

Background: We used Ripley’s K functions to test the point patterns of participating and non-participating households against the null hypothesis of “random labelling.” Rather than the traditional null hypothesis of complete spatial randomness (CSR), testing against random labelling allows for the comparison of marks (or labels) within a heterogeneous system. This approach is useful in epidemiologic settings when human populations are assumed to be heterogeneous and the research question involves testing for non-random allocation of labels (e.g., cases and controls, participants and non-participants) within that environment. Traditionally, Ripley’s K function tests for departures from CSR by comparing the observed and expected number of events (i.e., points) within an expanding distance (*d*) from each point. Diggle [1] and Dixon [2], however, proposed that Ripley’s K functions could be used to test against the random labelling hypothesis by comparing the difference in Ripley’s K functions between cases and controls. They proposed that no spatial dependence would exist if:

K_11_(d) = K_22_(d) = K_12_(d) = K_21_(d)

…where K_11_(d) represents the K function for clustering among cases (i.e., the observed average number of cases within a distance *d* of each case), K_22_(d) represents the same K function for clustering among controls, and K_12_(d) and K_21_(d) represent the cross-K functions (i.e., the observed average number of controls within a distance *d* of each case, and vice versa).

Methods: In this analysis, we tested for the random labelling of participants and non-participants using the K1K2 function in R, which is a function available through the “Spatstat” and “Ececpa” R packages. We defined participating households (K_1_) as those in which all household members provided stool samples, and non-participating households (K_2_) as those in which at least one member refused or was unavailable to provide a stool sample. The K1K2 function provides three graphical outputs to test the random labelling hypothesis: K_11_(d) – K_22_(d), K_11_(d) – K_12_(d), and K_22_(d) – K_12_(d). Each plot is accompanied with a 95% significance envelope, which was produced by running 1000 Monte Carlo simulations. If the observed K value (red line) runs outside of the envelope, it is indicative of non-random labelling at that distance (i.e., significant clustering of participants or non-participants, depending on the plot). The three K1K2 plots were produced for each of the seven villages in this study.

Results: 4 of the 7 villages analyzed did not show any significant non-random patterns in the distribution of participant and non-participant households, indicating that participation in these villages is unlikely to have been related to spatial processes that could have biased the observed spatial relationships in this study. In one village (Algodonal), the K_11_ – K_12_ line ran below the 95% significance envelope, indicating a trend toward non-random uniformity of participation. In two villages (Cachaco and Buenos Aires), the K_22_ – K_12_ line ran above the 95% significance envelope, indicating that there was significant clustering of non-participant households. The clustering occurred at distances of 250-275 meters (Cachaco) and 30 meters (Buenos Aires). Evaluating the maps of these villages reveals a few tight clusters of non-participating households dispersed throughout Buenos Aires (30m clustering), and a wider trend of non-participating households in the south end of Cachaco (250-275m clustering).

Conclusions: If clustered non-participant households were at greater/lesser risk for *T. solium* taeniasis (due to rurality, geographic trends in pig rearing, diet, socio-economic status, etc.), our estimates for the prevalence of taeniasis in these villages could be biased, and calculated distances between infected pigs and tapeworm carriers could be less accurate. It is unlikely, however, that these small detected clusters would have significantly impacted our results. Further, any spatial participation bias would have only exaggerated observed distance values, causing an attenuation in the effect of proximity to tapeworm carriers on the odds of porcine cysticercosis observed in this analysis.

References:

1. Diggle PJ. Point process modelling in environmental epidemiology. In: Barnett V, Turkman K, editors. Statistics for the environment. Chichester: Wiley; 1993.

2. Dixon PM. Ripley’s K function. In: El-Shaarawi AH, Piergorsch WW, editors. Encyclopedia of environmetrics. Chichester: Wiley; 2002. pp. 1796–1803.

Figures:

**Village: Cachaco (507)**


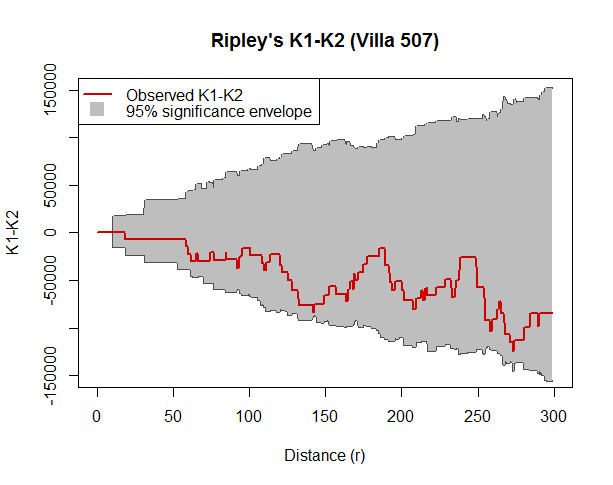

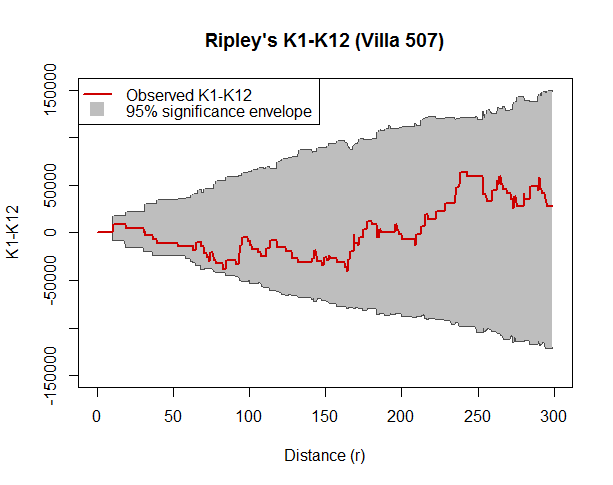


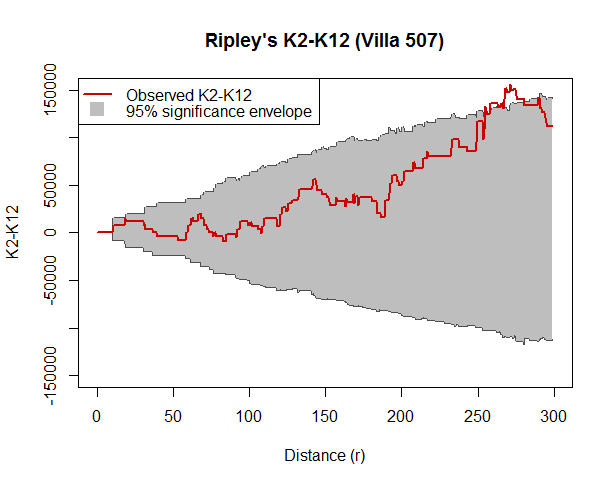


**Village: La Saucha (510)**


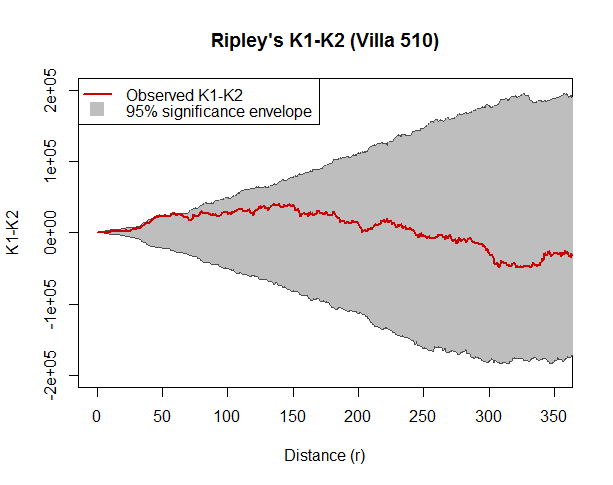

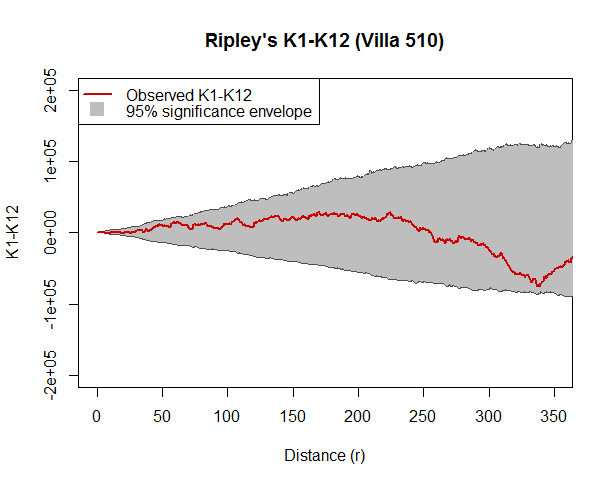


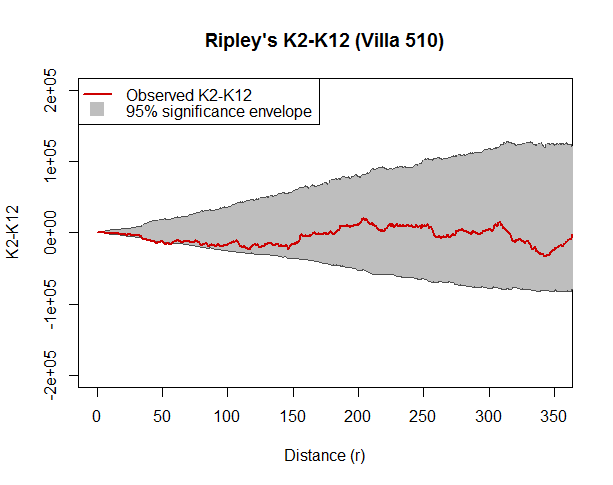


**Village: Tomapampa de Cardal (515)**


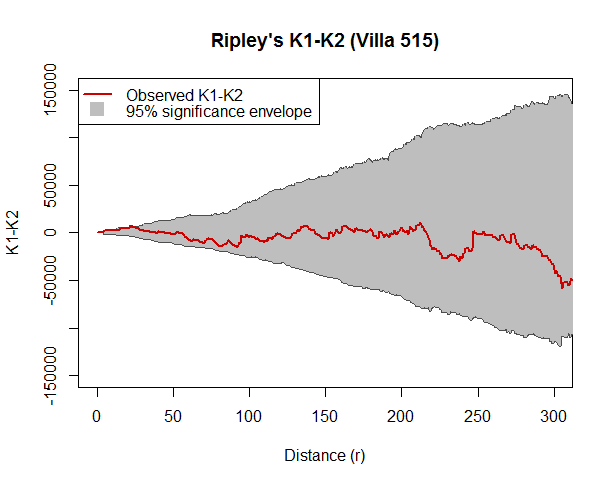

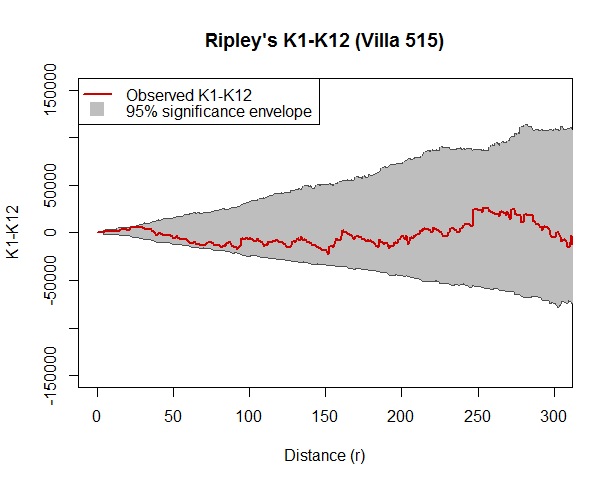


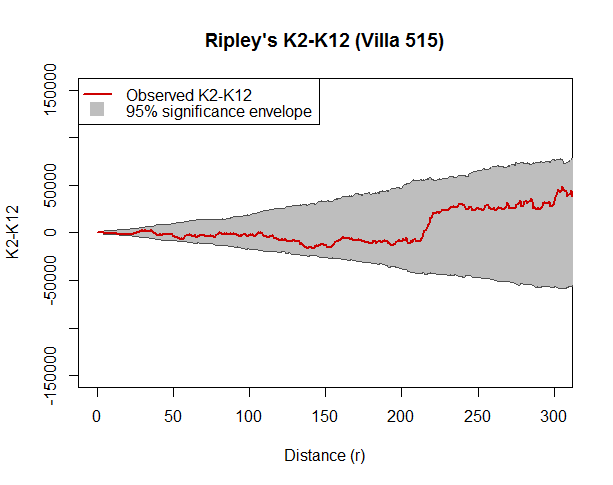


**Village: Puente Quiroz (566)**


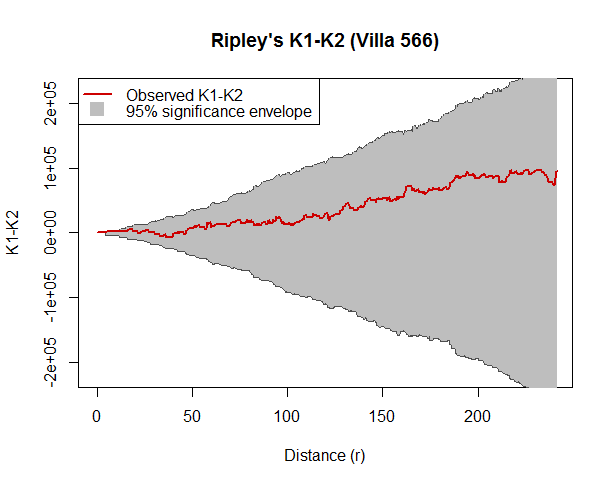

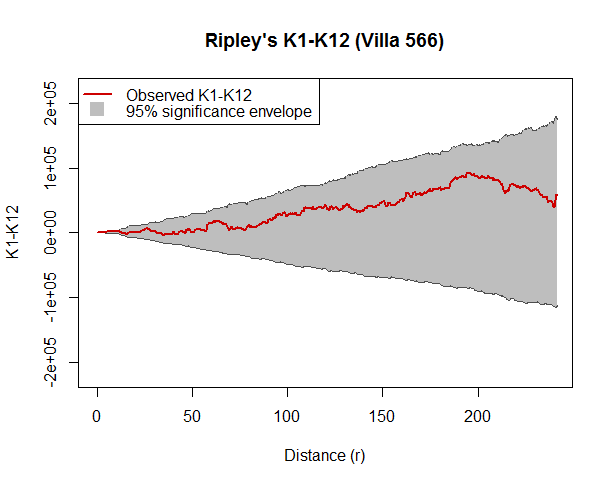


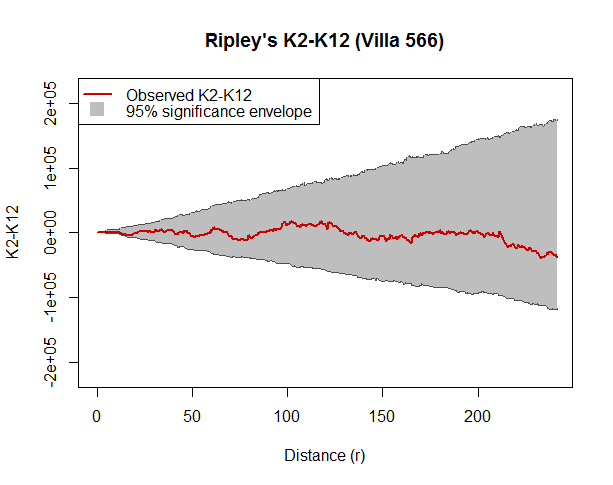


**Village: Culqui (567)**


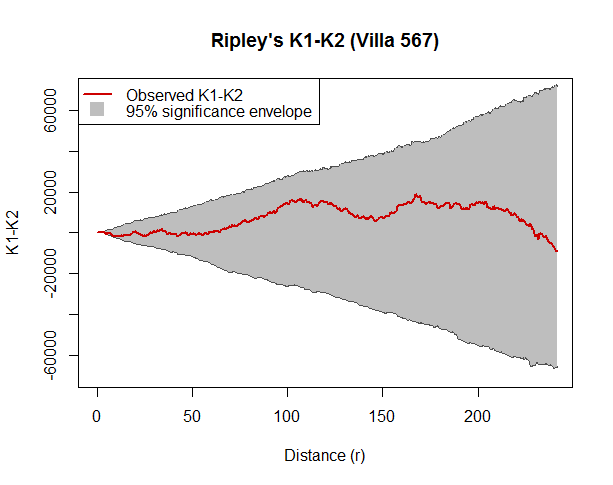

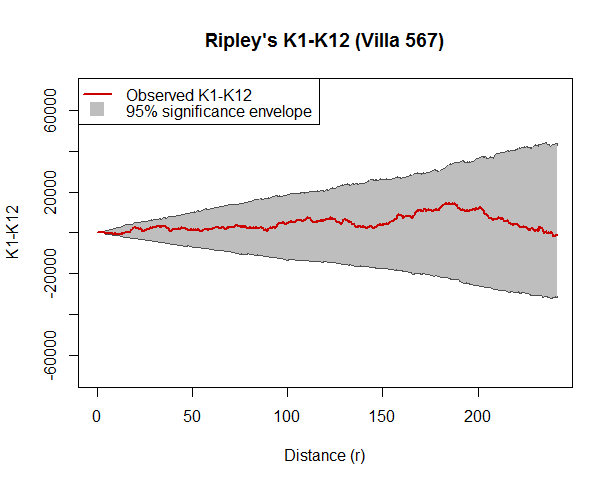


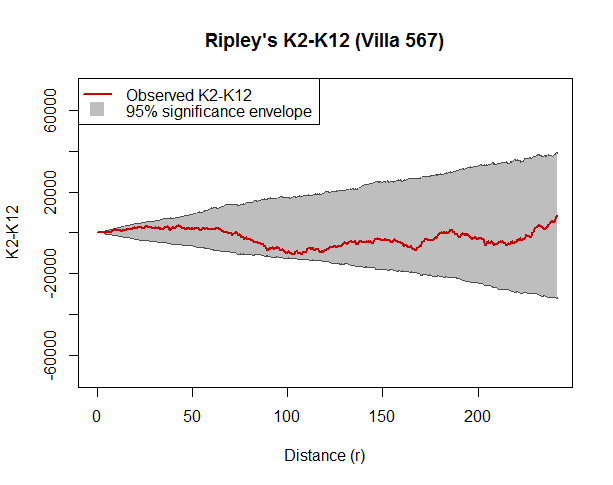


**Village: Algodonal (568)**


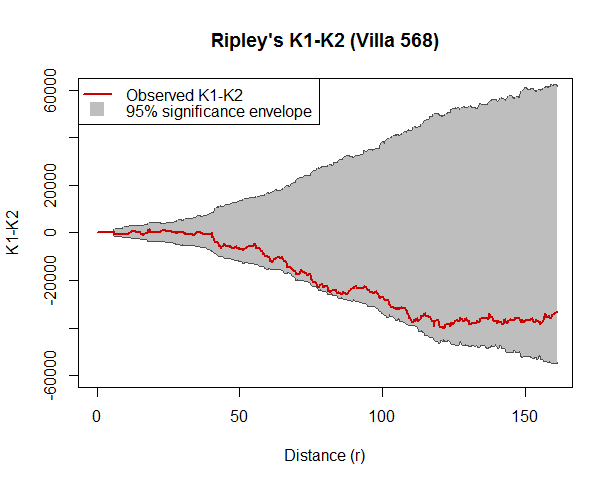

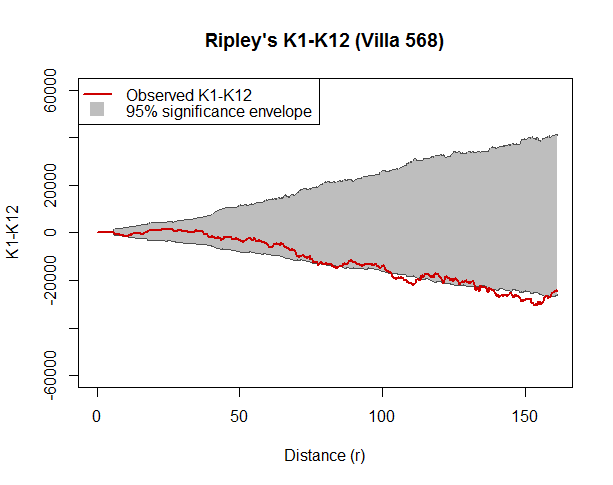


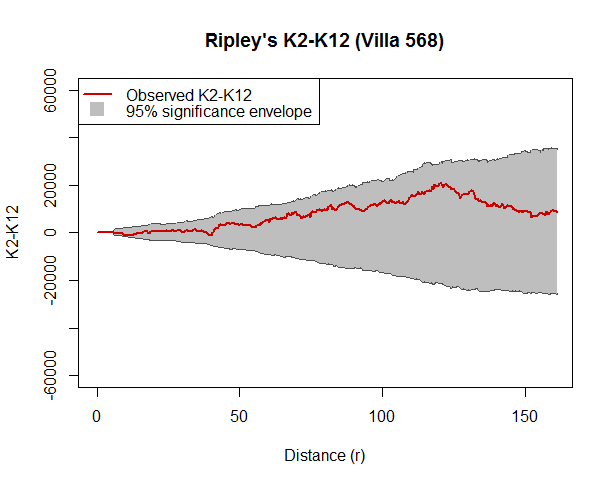


**Village: Buenos Aires (569)**


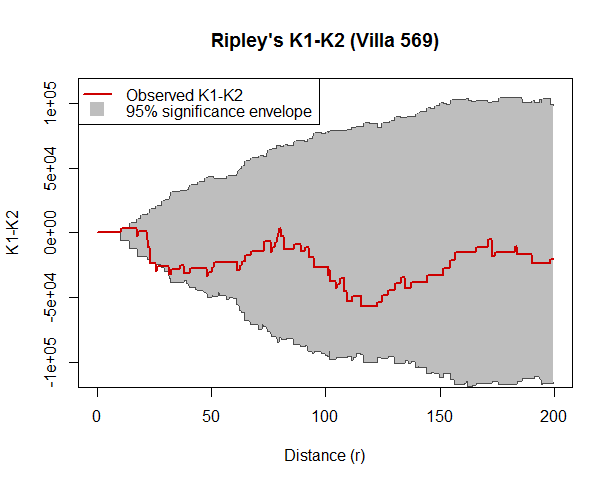

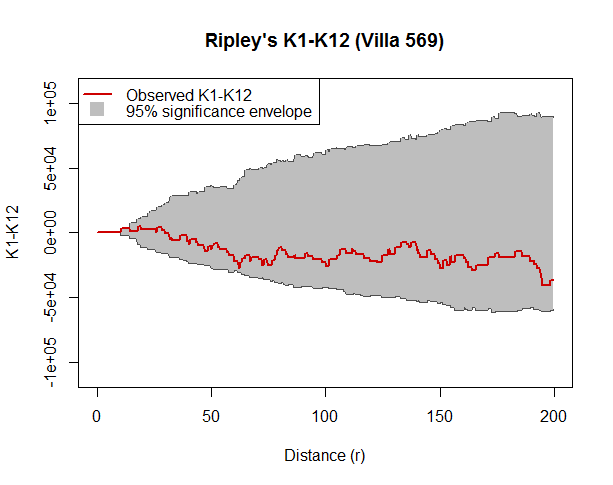


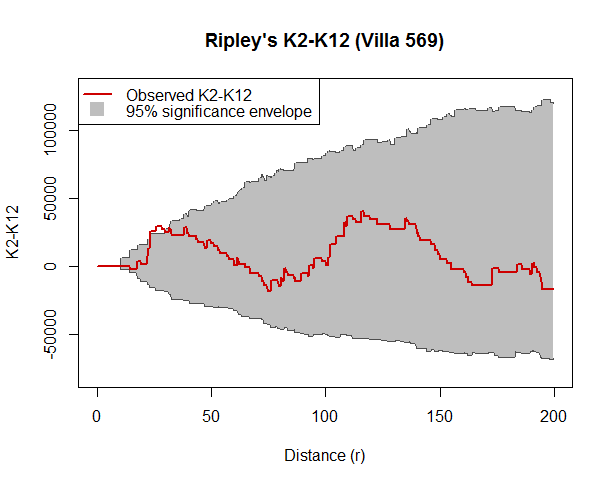

Supplement: S1 Appendix — (DOCX) [file pntd.0005536.s003.docx]
