# Supplementary material for: Spatial relationship between Taenia solium tapeworm carriers and necropsy cyst burden in pigs
Source: PLoS Negl Trop Dis. 2017 Apr 13;11(4):e0005536. doi: 10.1371/journal.pntd.0005536 (PMC5404875; doi:10.1371/journal.pntd.0005536)
Supplement: S2 Appendix — (DOCX) [file pntd.0005536.s004.docx]

**S2 Appendix. Sensitivity analysis comparing distance associations using different case definitions for CoAg-ELISA positivity (human).**

Case definition used (*T. solium* taeniasis):

- Co-Ag ELISA ODR≥20%
- *Taenia spp.* eggs identified in stool (microscopy)

Of the **34** cases identified:

- 26 (76%) had ODR ≥ 40% (these will be referred to as “likely” cases)
- 8 (24%) had ODR < 40% (these will be referred to as “probable” cases)

Of the **8** probable cases:

- 2 (25%) were identified by microscopy alone (ODR=14%,16%)
- 4 (50%) had both positive microscopy and ODR ≥ 20% (ODR=23%, 24%, 33%, 36%)
- 2 (25%) had negative microscopy and ODR ≥ 20% (ODR=30%, 34%)

*­*

Prevalence of porcine cysticercosis surrounding “likely” and “probable” cases of *T. solium* taeniasis, based on Co-Ag ELISA results:

*Probable cases (n=8)*

- Prevalence of porcine cysticercosis (≥1 cyst) in pigs within 50m = **14%** (5/36)
- Prevalence of porcine cysticercosis (≥100 cysts) in pigs within 50m = **6%** (2/36)

*Likely cases (n=26)*

- Prevalence of porcine cysticercosis (≥1 cyst) in pigs within 50m = **17%** (7/41)
- Prevalence of porcine cysticercosis (≥100 cysts) in pigs within 50m = **7%** (3/41)

*Two-sample test of proportions*:*

- ≥1 cyst:
  - 14% surrounding “probable” cases
  - 17% surrounding “likely” cases
  - **p-value = 0.76**
- ≥100 cysts: p-value=0.75 (6% vs. 7%)
  - 6% surrounding “probable” cases
  - 7% surrounding “likely” cases
  - **p-value = 0.99**

*tests for significant differences in the prevalence of porcine cysticercosis surrounding “likely” and “probable” cases of *T. solium* taeniasis using Fisher’s exact test of two binomial proportions.

Conclusion: Cases of *T. solium* taeniasis that were detected at different CoAg-ELISA detection thresholds had similar spatial association with infected pigs (≥1 cyst and ≥100 cysts) in this study. While it is possible that some tapeworm carriers identified in this study represented false positive diagnoses (due to non-specific binding and cross-reaction of the CoAg-ELISA assay with other *Taenia spp.*), it is unlikely that these false positives, if present, significantly affected the spatial associations observed in this study.
